# Autologous macrophages stimulated by epoxides of polyunsaturated fatty acids and protected by inhibitors of soluble epoxide hydrolase or cGAS/STING pathway repair amyloid‐β pathology in the Alzheimer’s disease (AD) brain

**DOI:** 10.1002/alz.085616

**Published:** 2025-01-03

**Authors:** Milan Fiala, Julian Whitelegge, Bruce D. Hammock, Santosh Kesari

**Affiliations:** ^1^ UCLA, Los Angeles, CA USA; ^2^ University of California, Los Angeles, Los Angeles, CA USA; ^3^ University of California, Davis, Davis, CA USA; ^4^ Pacific Brain Health Center, Pacific Neuroscience Institute Foundation, Santa Monica, CA USA

## Abstract

**Background:**

Brain accumulation of amyloid‐ß_1‐42_ (Aß) in plaques and neurons is the cause of AD neuropathology that is opposed by autologous monocyte/macrophages (MMs) in health but this defense fails in AD.

**Method:**

RNAseq, immunochemistry of the brain, immunofluorescence, and confocal microscopy of macrophages.

**Result:**

In the AD brain, MMs shuttle Aß from parenchyma to vessels, which develop vasculitis, causing amyloid‐related imaging abnormalities (ARIAs). AD patients’ MMs are inflammatory and lack enzymes in energy, and chaperones for unfolded protein response (UPR) and Aß degradation. Epoxides of polyunsaturated fatty acids (EpFAs), in combination with the inhibitors of soluble epoxide hydrolase (sEH) TPPU or EC5026 or the cGAS/STING pathway, regulate in macrophages in a homeostatic fashion Aβ‐degrading enzymes, inflammatory cytokines, and unfolded protein response (UPR) transcripts, and recover a pro‐resolution macrophage phenotype. The repaired macrophages display increased phagocytosis of FITC‐ Aß at 2 hours and increased degradation of Aβ at 24 hours. Others showed in a mouse model that restoration of cellular bioenergetics through inhibition of the prostaglandin E2 (PGE2) receptor 4 (EP4R) signaling restored cognition.

**Conclusion:**

Inhibiting macrophage inflammation by the sEH receptor inhibitors TPPU and EC5026 or the STING inhibitor H‐151 in AD macrophages could restore cognition in AD patients.

